# Sex-Specific Associations of α-Synuclein Pathology With Tau Accumulation

**DOI:** 10.1001/jamanetworkopen.2026.0461

**Published:** 2026-03-04

**Authors:** Elijah Mak, Angela J. Fought, Heather J. Wiste, Scott A. Przybelski, Robert I. Reid, Christopher G. Schwarz, Matthew L. Senjem, Prashanthi Vemuri, Clifford R. Jack, Val J. Lowe, Ronald C. Petersen, Walter A. Rocca, Bradley F. Boeve, Kejal Kantarci

**Affiliations:** 1 Department of Radiology, Mayo Clinic, Rochester, Minnesota; 2 Department of Quantitative Health Sciences, Mayo Clinic, Rochester, Minnesota; 3 Department of Information Technology, Mayo Clinic, Rochester, Minnesota; 4 Department of Neurology, Mayo Clinic, Rochester, Minnesota; 5 Division of Epidemiology, Department of Quantitative Health Sciences, Mayo Clinic, Rochester, Minnesota; 6 Women’s Health Research Center, Mayo Clinic, Rochester, Minnesota

## Abstract

**Importance:**

Sex differences are increasingly recognized as modifiers of Alzheimer disease and related dementias, with women exhibiting greater tau burden and faster cognitive decline than men. Even though α-synuclein copathology frequently occurs in Alzheimer disease, its contribution to sex differences in disease progression is unclear.

**Objective:**

To test whether α-synuclein positivity, measured using cerebrospinal fluid seed amplification assay (SAA), is differentially associated with tau accumulation in women vs men across the Alzheimer disease continuum.

**Design, Setting, and Participants:**

This cohort study used longitudinal tau positron emission tomography from the Alzheimer’s Disease Neuroimaging Initiative collected between 2015 and 2023, with a median (IQR) follow-up of 1.23 (0.00-3.84) years. Participants were stratified by cerebrospinal fluid α-synuclein seed amplification assay status and sex. Participants were cognitively unimpaired or cognitively impaired (mild cognitive impairment or dementia) at baseline.

**Exposure:**

Cerebrospinal fluid α-synuclein status determined by SAA and dichotomized as SAA negative or SAA positive.

**Main Outcomes and Measures:**

Tau burden was quantified as standardized uptake value ratio (SUVr) in the medial temporal composite region of interest. Linear mixed-effects models tested SAA by sex by time interactions on longitudinal tau accumulation, adjusting for baseline age, baseline cognitive status, apolipoprotein E ε4 carrier status, and site. Sample size estimates were calculated to detect 25% and 50% treatment effects with 80% power in those with cognitive impairment.

**Results:**

Among 415 participants (mean [SD] age, 72.3 [7.6] years; 220 women [53%]; 69 SAA positive [17%] and 346 SAA negative [83%]), there was a significant interaction between SAA status, sex, and time on tau accumulation (β, 0.061; 95% CI, 0.030-0.093; *P* < .001). Women with positive SAA results exhibited the fastest tau accumulation compared with other groups (0.066 SUVr per year; 95% CI, 0.043 to 0.089 SUVr per year; *P* < .001). Clinical trials targeting tau pathology in cognitively impaired individuals with 18-month follow-up would require 129 SAA-positive women to detect a 25% treatment effect with 80% power, compared with 518 SAA-negative women.

**Conclusions and Relevance:**

In this cohort study of participants across the Alzheimer disease continuum, α-synuclein copathology was associated with faster tau accumulation in women than men. These findings may inform sex-specific interpretation of α-synuclein biomarkers and trial design.

## Key Points

**Question:**

Is α-synuclein pathology differentially associated with tau accumulation in women and men across the Alzheimer disease continuum?

**Findings:**

In this cohort study of 415 participants from the Alzheimer’s Disease Neuroimaging Initiative who underwent longitudinal tau positron emission tomography, women with positive α-synuclein seed amplification assay results had significantly faster tau accumulation than all other groups, whereas men with positive results did not differ significantly from men with negative results.

**Meaning:**

These findings suggest that α-synuclein copathology in women is associated with faster tau accumulation, supporting sex-specific biomarker interpretation and trial stratification in Alzheimer disease.

## Teaser

This cohort study examines whether α-synuclein positivity is associated with tau accumulation in women vs men across the Alzheimer disease continuum.

## Introduction

Women account for two-thirds of Alzheimer disease (AD) cases, exhibiting greater tau pathology, worse cognitive decline, and increased apolipoprotein E ε4 allele (APOE ε4) susceptibility compared with men.^1^ Similar sex differences extend to dementia with Lewy bodies (DLB), where women show distinct atrophy patterns and greater AD copathology.^2^

α-Synuclein, the neuropathological hallmark of Lewy body diseases, co-occurs with AD pathology in up to 50% of cases.^3^ Cerebrospinal fluid (CSF) α-synuclein seed amplification assays (SAAs) now enable in vivo detection of misfolded α-synuclein with greater than 90% diagnostic accuracy.^4,5^ In AD cohorts, SAA positivity associates with faster neurodegeneration and cognitive decline independent of AD biomarkers.^6,7,8^

However, whether α-synuclein copathology is differentially associated with disease progression by sex remains unexplored. Prior research has often treated sex as a statistical covariate rather than a biological modifier of α-synuclein effects, creating a knowledge gap with important clinical implications for biomarker interpretation and therapeutic development. This knowledge gap has important clinical implications, as sex-specific pathological interactions are not currently incorporated into biomarker interpretation or therapeutic development strategies. We leveraged longitudinal tau positron emission tomography (PET) and CSF SAA data from the Alzheimer’s Disease Neuroimaging Initiative (ADNI) to determine whether α-synuclein pathology is associated with differences in the rate of tau accumulation in women and men.

## Methods

### Participants

This cohort study utilized ADNI data from participants with both CSF SAA results and tau PET imaging. The sample included cognitively unimpaired individuals and those with cognitive impairment (mild cognitive impairment or dementia). Standard ADNI inclusion or exclusion criteria applied. Per ADNI protocol, participants with Parkinson disease or other serious neurological diseases were excluded. The ADNI study was approved by each ADNI study site’s respective institutional review board, and informed written consent was obtained from all participants. This cohort study is reported in accordance with the Strengthening the Reporting of Observational Studies in Epidemiology (STROBE) reporting guidelines for cohort studies.

### CSF α-Synuclein SAA

Detailed methods for CSF collection, processing, and the α-synuclein SAA technique have been described previously.^4^ CSF was collected per ADNI protocols and analyzed at Amprion Clinical Laboratory using clinically validated methods. The α-synuclein SAA has been validated against postmortem neuropathological examination, demonstrating 97.8% sensitivity and 98.1% specificity for detecting limbic and/or neocortical Lewy body pathology and 100% sensitivity and 96.3% specificity for diffuse and transitional Lewy body disease in autopsy-confirmed cases.^9,10^ Only detected type 1 cases were considered SAA positive, and only not detected cases were SAA negative.

### PET Imaging

^18F^Flortaucipir PET scans followed standardized ADNI protocols with 30-minute acquisitions 75 to 105 minutes after injection.^11^ The primary outcome was a metatemporal composite standardized uptake value ratio (SUVr) comprising the bilateral entorhinal cortex, amygdala, fusiform gyrus, and inferior or middle temporal cortices, referenced to cerebellar gray matter.

### Statistical Analysis

To test our primary hypothesis that α-synuclein pathology is differentially associated with tau accumulation rates in men vs women, we fit linear mixed-effects models of metatemporal tau PET SUVR with fixed effects for time, SAA status, and sex; all 2-way interactions among these variables (SAA by sex, SAA by time, and sex by time); and the 3-way interaction of time by SAA by sex. The model was also adjusted for baseline age, baseline cognitive status, APOE ε4 carrier status, and site, with a random intercept for participant to account for repeated measures. Next, we estimated group-specific tau accumulation slopes using the estimated marginal trends (emmeans package in R statistical software version 4.5.1; R Project for Statistical Computing). Pairwise comparisons between groups were performed, with *P* values adjusted for multiple comparisons using the false discovery rate (FDR) method. We also estimated the number of participants per group required to detect 25% and 50% treatment effects on tau accumulation with 80% power and 1-sided α = .05, across trial durations of 12, 18, 24, and 36 months in participants with cognitive impairment (mild cognitive impairment and dementia). Statistical significance was defined as 2-sided *P* < .05.

## Results

### Participant Characteristics

Data were collected between 2015 and 2023, with a median (IQR) follow-up of 1.23 (0.00-3.84) years. A total of 415 participants were included (mean [SD] age, 72.3 [7.6] years; 220 women [53%]; 69 SAA positive [17%] and 346 SAA negative [83%]; 172 APOE-ε4 carriers [41%]; 201 cognitively impaired at baseline [48%]). Baseline characteristics are summarized in Table 1. Baseline characteristics of cognitively unimpaired participants are shown in the eTable in Supplement 1.

**Table 1.** Baseline Characteristics of Sample, Stratified by SAA Status and Biological Sex

Characteristic	Patients, No. (%)				*P* value
	SAA negative		SAA positive		
	Male (n = 153)	Female (n = 193)	Male (n = 42)	Female (n = 27)	
Age at baseline, mean (SD), y	73.8 (7.7)	70.1 (7.3)	76.4 (6.5)	73.3 (7.3)	<.001
Apolipoprotein E ε4 allele positive	53 (35)	83 (43)	19 (45)	17 (63)	.04
Baseline cognition: cognitively impaired	90 (59)	70 (36)	23 (55)	18 (67)	<.001
Clinical Dementia Rating–Sum of Boxes score, mean (SD)	1.27 (1.86)	0.75 (1.55)	1.83 (2.28)	2.20 (2.46)	<.001
Tau-PET metatemporal standardized uptake value ratio, mean (SD)	1.31 (0.31)	1.28 (0.26)	1.36 (0.35)	1.59 (0.47)	.001
Abnormal amyloid-PET	67 (46)	78 (42)	23 (59)	20 (77)	.004
Follow-up, median (IQR), y	1.01 (0.00-3.70)	1.97 (0.00-3.96)	1.08 (0.00-3.62)	1.01 (0.00-2.26)	.60

Abbreviations: PET, positron emission tomography; SAA, α-synuclein seeding amplification assay.

### SAA by Sex by Time Interactions on Tau PET

Raw tau-PET data points with within-subject lines are shown in panel A of the Figure. We observed a statistically significant 2-way interaction between SAA status and sex (β, 0.168; 95% CI, 0.006 to 0.329; *P* = .04). The 3-way interaction between SAA status, sex, and time was also statistically significant, indicating that the rate of tau accumulation over time differed by both SAA status and sex (β, 0.061; 95% CI, 0.030 to 0.093; *P* < .001) (Figure). Panel B of the Figure displays model-estimated trajectories from the fully adjusted interaction model, showing that SAA-positive women exhibited the steepest increase in tau accumulation. Group-specific slope estimates in panel C of the Figure, together with adjusted pairwise contrasts, confirmed that SAA-positive women accumulated tau significantly faster than SAA-negative men, SAA-negative women, and SAA-positive men (all FDR-adjusted *P* < .05). Estimated tau accumulation rates were 0.013 SUVr per year (95% CI, 0.004 to 0.022 SUVr per year; *P* = .004) for SAA-negative men, 0.015 SUVr per year (95% CI, 0.008 to 0.023 SUVr per year; *P* < .001) for SAA-negative women, 0.003 SUVr per year (95% CI, −0.015 to 0.020 SUVr per year; *P* = .76) for SAA-positive men, and 0.066 SUVr per year (95% CI, 0.043 to 0.089 SUVr per year; *P* < .001) for SAA-positive women. In contrast, SAA-positive men did not differ significantly from SAA-negative individuals of either sex. Sample size estimates for hypothetical clinical trials targeting tau accumulation in cognitively impaired individuals are presented in Table 2. For an 18-month trial designed to detect a 25% treatment effect with 80% power, SAA-positive women would require 129 participants per group, compared with 518 for SAA-negative women, 2033 for SAA-negative men, and 10 247 for SAA-positive men.

**Figure.**
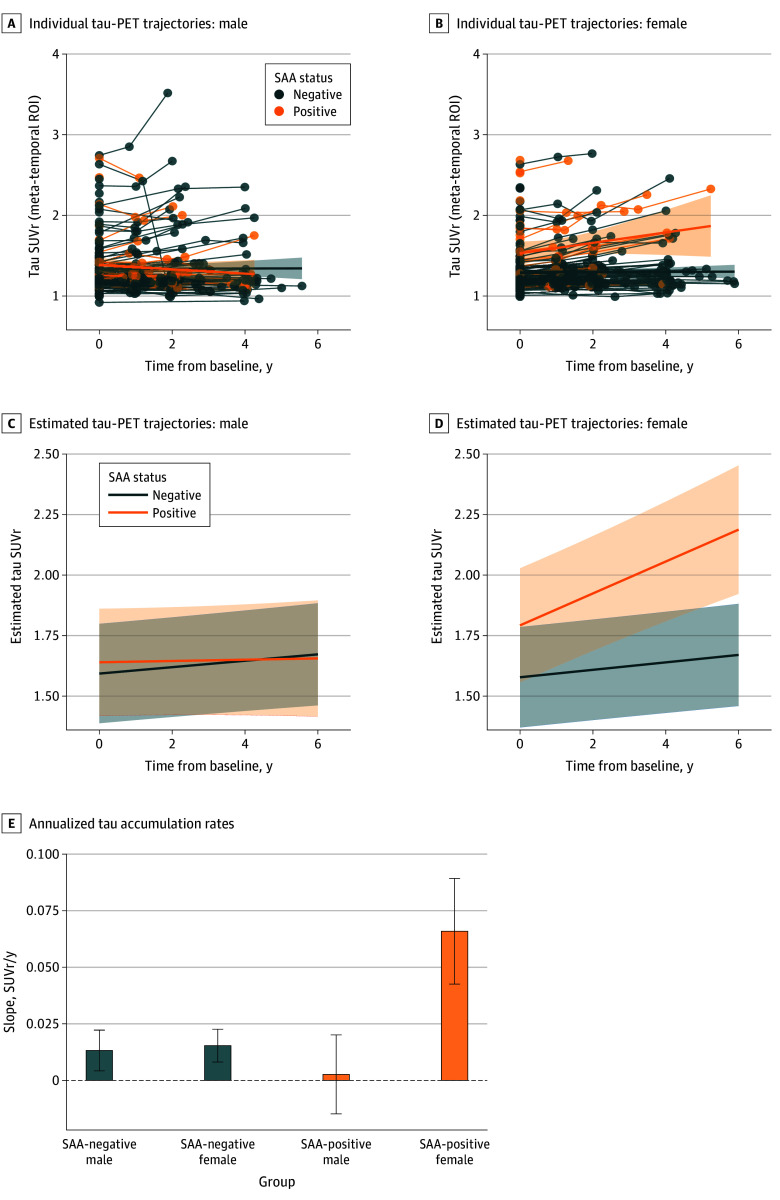
Graphs of Longitudinal Tau Accumulation by Sex and α-Synuclein Seeding Amplification Assay (SAA) Status A and B, Observed participant trajectories of tau standardized uptake value ratio (SUVr) across time for male (A) and female (B) participants. Overlaid ordinary least-squares group trends by SAA and sex with 95% CI are shown for visualization only and were not used for inference. C and D, Model-based estimated trajectories from the 3-way linear mixed-effects model (SAA by sex by time) adjusted for apolipoprotein E ε4 allele carrier status, site, baseline cognition status and age at baseline are shown for male (C) and female (D) patients. Shaded areas show 95% CIs. E, Model-estimated slopes (annualized tau-accumulation rates, SUVr per year) were derived from the SAA by sex by time interaction in the same mixed-effects model. The error bars show 95% CIs. PET indicates positron emission tomography; ROI, region of interest.

**Table 2.** Sample Size Estimates Per Group for Clinical Trials Targeting Tau Accumulation in Cognitively Impaired Individuals^a^

Sample	12 Months		18 Months		24 Months		36 Months	
	25% Effect	50% Effect	25% Effect	50% Effect	25% Effect	50% Effect	25% Effect	50% Effect
SAA negative								
Male	4586	1138	2033	505	1145	286	509	128
Female	1159	291	518	129	293	72	130	32
SAA positive								
Male	22 638	5874	10 247	2600	5665	1456	2631	670
Female	295	73	129	32	73	18	33	9

Abbreviation: SAA, α-synuclein seeding amplification assay.

^a^ Sample sizes 2343 calculated using longitudinal power analysis for 2-group trials with 80% power and 1-sided α = .05.

### Sensitivity Analyses

We performed a sensitivity analysis including baseline tau-PET SUVR as a covariate to determine whether the accelerated accumulation was independent of baseline tau differences. The 3-way interaction persisted after baseline tau adjustment (β, 0.064; 95% CI, 0.039-0.089; *P* < .001), with SAA-positive women accumulating tau faster than SAA-negative women (β, 0.054; 95% CI, 0.035-0.073; FDR-adjusted *P* < .001). A second sensitivity analysis restricted to 214 cognitively unimpaired participants with adjustment for baseline tau-PET SUVr was performed to ensure findings were not mediated by disease severity. The 3-way interaction between SAA status, sex, and time remained significant (β, 0.054; 95% CI, 0.035-0.073; *P* < .001). Individual tau trajectories for cognitively unimpaired participants are shown in panel A of the eFigure in Supplement 1, with model-estimated group trajectories and 95% CIs in panel B of the eFigure in Supplement 1.

## Discussion

In this cohort study, we demonstrated that α-synuclein pathology is selectively associated with accelerated tau accumulation in women, with SAA-positive women showing more than 20-fold higher accumulation rates than SAA-positive men. These findings extend prior evidence of female susceptibility in AD^12^ and DLB^13^ by identifying α-synuclein as a pathological catalyst that selectively affects women. It has been reported that SAA-positive individuals are likely to have higher baseline tau burden,^7^ raising the question of whether the accelerated tau accumulation observed in SAA-positive women simply reflects their higher starting point rather than a true longitudinal outcome. Our sensitivity analysis addressed this by adjusting for baseline tau-PET SUVr, demonstrating that α-synuclein copathology may confer a sex-specific vulnerability to tau progression superimposed on baseline differences.

Several mechanisms may underlie this vulnerability. Estrogen depletion compromises neuroprotective autophagy and microglial regulation,^14^ leading to permissive environments for protein aggregation. Our group recently demonstrated that bilateral oophorectomy performed before the age of 46 years increases tau burden in women with elevated amyloid.^15^ Against this backdrop of depleted neuroprotection, we propose that α-synuclein pathology may represent a second hit amplifier that exploits these existing vulnerabilities likely through cross-seeding tau aggregation,^16^ as well as amplified neuroinflammation that drives tau phosphorylation.^17^ These findings may inform precision medicine approaches in AD, such that α-synuclein SAA status, considered by sex, could be incorporated into sample size estimation and stratification in trials targeting tau or α-synuclein pathology. For instance, our analyses showed that an 18-month trial would require only 129 participants per group to detect a 25% treatment effect with 80% power, compared with 518 SAA-negative women per group (ie, a 75% reduction in sample size).

Another important implication of our findings relates to diagnostic accuracy in clinical practice. DLB is less common in women than men; however, when DLB does occur in women, it is typically accompanied by substantial AD copathology.^13^ As a result, α-synuclein pathology in women may be systematically underdetected because of masking by more overt AD features, with women more likely to receive an AD diagnosis despite harboring substantial synuclein burden. Without specific biomarkers such as SAA, this diagnostic masking could lead to suboptimal management of Lewy body symptoms and even exclusion of women from disease-modifying clinical trials.

### Limitations

This study has limitations that should be mentioned. The SAA-positive female sample of 27 participants is limited, and replication in independent cohorts with larger samples of SAA-positive women is essential to confirm these findings and explore mechanisms underlying this sex-specific effect. Binary SAA classification prevented assessment of dose-dependent effects and quantitative comparisons of α-synuclein pathology burden across the full cohort. In addition, the absence of a primary DLB comparison cohort precluded direct comparison of pathology burden levels between AD plus Lewy body and primary Lewy body disease, although all SAA-positive participants showed Parkinson disease and/or DLB-like seeding patterns identical to those observed in clinically diagnosed DLB. Future studies incorporating quantitative α-synuclein measures could enable continuous modeling approaches utilizing bigger sample sizes. The relatively short follow-up time frame may not capture delayed effects in men. ADNI is primarily composed of individuals on the AD spectrum (cognitively unimpaired or with AD-related cognitive impairment). By design, participants with prominent DLB clinical features were excluded, and ADNI does not collect measures of parkinsonism (eg, the Unified Parkinson Disease Rating Scale) or dopaminergic neuronal integrity (eg, dopamine transporter scan). Therefore, we could not determine whether α-synuclein SAA-positive participants had extrapyramidal symptoms or underlying nigrostriatal dopaminergic degeneration, and some individuals may have had prodromal DLB or mixed pathology. As such our findings may therefore not generalize to primary DLB cohorts, in which α-synuclein pathology and parkinsonism are core clinical features rather than copathology.

## Conclusions

In this cohort study of cognitively unimpaired and cognitively impaired ADNI participants with CSF α-synuclein SAA results and longitudinal tau PET imaging, α-synuclein pathology was associated with sex-specific differences in tau accumulation over time. These findings emphasized sex as a biological variable that should be integrated into interpretation of α-synuclein and AD biomarkers and clinical trial design and future precision medicine approaches targeting sex-specific pathological pathways.

## Notes

Supplement 1. eTable. Baseline characteristics of cognitively unimpaired participants eFigure. Tau PET trajectories in cognitively unimpaired participants

Supplement 2. Nonauthor Collaborators. Alzheimer’s Disease Neuroimaging Initiative Collaborators

Supplement 3. Data Sharing Statement
