# Supplementary material for: Sex-Specific Associations of α-Synuclein Pathology With Tau Accumulation
Source: JAMA Netw Open. 2026 Mar 4;9(3):e260461. doi: 10.1001/jamanetworkopen.2026.0461 (PMC12961516; doi:10.1001/jamanetworkopen.2026.0461)
Supplement: Supplement 1. — eTable. Baseline characteristics of cognitively unimpaired participants eFigure. Tau PET trajectories in cognitively unimpaired participants [file jamanetwopen-e260461-s001.pdf]

Supplemental Online Content

Mak E, Fought AJ, Wiste HJ, et al; Alzheimer's Disease Neuroimaging Initiative. Sex-specific associations of α-synuclein pathology with tau accumulation. *JAMA Netw Open*. 2026;9(3):e260461. doi:10.1001/jamanetworkopen.2026.0461

eTable. Baseline characteristics of cognitively unimpaired participants

eFigure. Tau PET trajectories in cognitively unimpaired participants

This supplemental material has been provided by the authors to give readers additional information about their work.

© 2026 Mak E et al. *JAMA Network Open*.

Characteristic	SAA-<br>Male<br>N = 63	SAA-<br>Female<br>N = 123	SAA+<br>Male<br>N = 19	SAA+<br>Female<br>N = 9	p-value
**Age at baseline, y**	74.35 ± 7.70	70.17 ± 6.52	77.57 ± 7.15	76.07 ± 5.29	<0.001
**APOE ε4 positive**					0.2
APOE4-	46 (73%)	73 (59%)	13 (68%)	7 (78%)	
APOE4+	17 (27%)	50 (41%)	6 (32%)	2 (22%)	
**CDR-SB**					>0.9
0	58 (92%)	110 (90%)	18 (95%)	8 (89%)	
0.5	4 (6.3%)	9 (7.4%)	1 (5.3%)	1 (11%)	
1	1 (1.6%)	3 (2.5%)	0 (0%)	0 (0%)	
Unknown	0	1	0	0	
**Tau-PET meta-temporal SUVr**	1.22 ± 0.20	1.20 ± 0.09	1.22 ± 0.10	1.29 ± 0.23	0.5
**Abnormal Amyloid-PET**	21 (34%)	43 (36%)	7 (39%)	5 (56%)	0.7
Unknown	2	2	1	0	
**Follow-up, y (median [IQR])**					0.5
Median [Q1, Q3]	1.01 [0.00, 4.01]	2.32 [0.00, 4.02]	3.93 [0.00, 4.05]	1.10 [0.00, 3.92]	

eTable. Baseline characteristics of cognitively unimpaired participants. Values are presented as mean ± standard deviation (SD) for continuous normally distributed variables, n (%) for categorical variables, and median [interquartile range, IQR]. Abbreviations: SAA, α-synuclein seeding amplification; APOE-ε4, apolipoprotein E ε4 allele; CU, cognitively unimpaired; CDR-SB, Clinical Dementia Rating – Sum of Boxes; SUVr, standardized uptake value ratio; IQR, interquartile range.

© 2026 Mak E et al. *JAMA Network Open*.

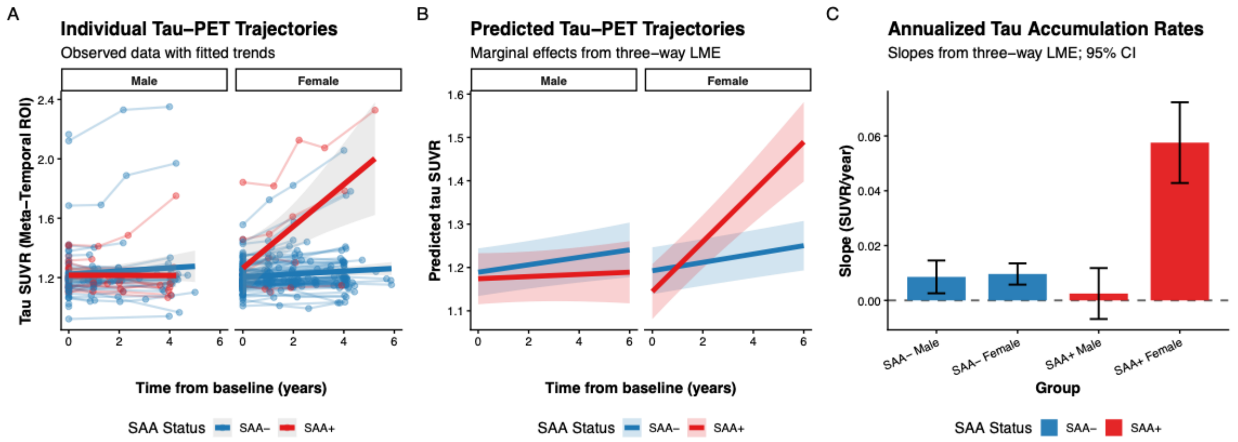

eFigure. Tau PET trajectories in cognitively unimpaired participants (A) Individual tau-PET trajectories for cognitively unimpaired participants, stratified by sex and SAA status. Thick lines represent group-level linear trends with 95% confidence intervals. Blue indicates SAA– participants; red indicates SAA+ participants. (B) Model-predicted tau-PET trajectories from mixed effects models adjusting for baseline tau-PET SUVR, APOE ε4 carrier status, age at baseline, and site. Abbreviations: SAA, α-synuclein seed amplification assay; SUVR, standardized uptake value ratio.

© 2026 Mak E et al. *JAMA Network Open*.
