# Supplementary material for: Sex-Specific Associations of α-Synuclein Pathology With Tau Accumulation
Source: JAMA Netw Open. 2026 Mar 4;9(3):e260461. doi: 10.1001/jamanetworkopen.2026.0461 (PMC12961516; doi:10.1001/jamanetworkopen.2026.0461)
Supplement: Supplement 2. — Nonauthor Collaborators. Alzheimer’s Disease Neuroimaging Initiative Collaborators [file jamanetwopen-e260461-s002.pdf]

*First name, last name, and suffix (if applicable) are required and will appear in PubMed.

***Group Name(s): Alzheimer's Disease Neuroimaging Initiative**

***First Name and Middle Initial(s)**	***Last Name**	***Suffix (eg, Jr, III)**	Academic Degrees	Institution	Location (city, state/province, country)	Role or Contribution, eg, chair, principal investigator	Group (if more than 1 Group listed in the byline) and/or Subgroup (eg, Steering Committee)
Michael W.	Weiner		M.D.	UCSF, NCIRE, VA Medical C	San Francisco, CA, U	Principal Investig	Alzheimer's Disease Neuroimaging Initiative (ADNI): Administrative Core
Paul	Aisen		M.D.	University of Southern Cali	Los Angeles, CA, US	Principal Investig	Alzheimer's Disease Neuroimaging Initiative (ADNI): Clinical Core / Coordinating Center
Ronald	Petersen		M.D., Ph	Mayo Clinic	Rochester, MN, USA	Principal Investig	Alzheimer's Disease Neuroimaging Initiative (ADNI): Clinical Core / Coordinating Center
Laurel	Beckett		Ph.D.	University of California, Davis	Davis, CA, USA	Principal Investig	Alzheimer's Disease Neuroimaging Initiative (ADNI): Biostatistics Core
Richard J.	Perrin		M.D., Ph	Washington University	St. Louis, MO, USA	Principal Investig	Alzheimer's Disease Neuroimaging Initiative (ADNI): Neuropathology Core
Arthur W.	Toga		Ph.D.	Laboratory of Neuro Imagi	Los Angeles, CA, US	Principal Investig	Alzheimer's Disease Neuroimaging Initiative (ADNI): Informatics Core
Andrew J.	Saykin		PsyD	Indiana University School o	Indianapolis, IN, US	Principal Investig	Alzheimer's Disease Neuroimaging Initiative (ADNI): Genetics Core
