# Supplementary material for: Sex-Specific Associations of α-Synuclein Pathology With Tau Accumulation
Source: JAMA Netw Open. 2026 Mar 4;9(3):e260461. doi: 10.1001/jamanetworkopen.2026.0461 (PMC12961516; doi:10.1001/jamanetworkopen.2026.0461)
Supplement: Supplement 3. — Data Sharing Statement [file jamanetwopen-e260461-s003.pdf]

Data Sharing Statement

Mak. Sex-Specific Associations of α-Synuclein Pathology With Tau Accumulation. *JAMA Netw Open*. Published March 04, 2026. doi:10.1001/jamanetworkopen.2026.0461

Data
Data available: Yes
Data types: Deidentified participant data
How to access data: Data used in preparation of this article were obtained from the ADNI database (adni.loni.usc.edu). As such, the investigators within the ADNI contributed to the design and implementation of ADNI and/or provided data but did not participate in analysis or writing of this report. A complete listing of ADNI investigators can be found at: http://adni.loni.usc.edu/wp-content/uploads/how_to_apply/ADNI_Acknowledgement_List.pdf
When available: With publication

Supporting Documents
Document types: None

Additional Information
Who can access the data: NA
Types of analyses: NA
Mechanisms of data availability: NA
Any additional restrictions: NA
